# Primary Healthcare Professionals' Perspectives on Cardiovascular Risk Communication to Persons With Type 2 Diabetes: A Grounded Theory Study

**DOI:** 10.1002/nop2.70828

**Published:** 2026-09-10

**Authors:** Anna‐Lena Stenlund, Karin Hellström Ängerud, Mikael Lilja, Julia Otten, Lena Jutterström

**Affiliations:** ^1^ Department of Nursing Umeå University Skellefteå Sweden; ^2^ Department of Nursing Umeå University Umeå Sweden; ^3^ Unit of Research, Education, and Development, Department of Public Health and Clinical Medicine Östersunds Hospital, Umeå University Umeå Sweden; ^4^ Family Medicine, Department of Public Health and Clinical Medicine Umeå University Umeå Sweden; ^5^ Section of Medicine, Department of Public Health and Clinical Medicine Umeå University Umeå Sweden

**Keywords:** cardiovascular risk, healthcare professionals' perspective, risk communication, type 2 diabetes

## Abstract

**Aim:**

To explore how cardiovascular risk communication is constructed, negotiated and enacted in practice to persons with type 2 diabetes.

**Design:**

Qualitative.

**Methods:**

Individual interviews were conducted with 14 HCPs in primary care. Data were analysed using the constructivist grounded theory method.

**Results:**

The core category, ‘Striving to enhance cardiovascular risk awareness in persons with type 2 diabetes while balancing responsibility and uncertainty’, captures the challenges in making CVD risk understandable and meaningful for patients. Four categories describe the HCPs' work: Navigating a complex assessment of cardiovascular risk, struggling to communicate cardiovascular risk in a pedagogical way, trying to build a trusting relationship and simultaneously highlighting the disease severity, and experiencing loneliness and requesting organisational changes.

**Conclusions:**

Healthcare professionals (HCPs) recognised their significant responsibility for cardiovascular risk communication, but simultaneously experienced uncertainty about how to assess patients' individual CVD risk. Diabetes specialist nurses described feelings of professional isolation, limited medical support from physicians and a lack of shared responsibility within the diabetes care team. HCPs also requested sufficient organisational resources.

**Implications for the Profession:**

Communicating cardiovascular risk remains difficult. Strengthening the role of HCPs in risk communication and ensuring they adopt a more person‐centred approach are essential to improving risk communication in primary healthcare.

**Impact:**

To assess cardiovascular risk and reveal patients' understanding, HCPs must go beyond education, build trust and understanding, and balance encouragement with demands.

**Reporting Method:**

COREQ checklist.

**Patient or Public Contribution:**

No patient or public contribution.

## 1 Introduction

Type 2 diabetes (T2D) is an escalating global health concern and accounts for 90% of the estimated 537 million individuals with diabetes worldwide (ADA 2026; Marx et al. 2023). Persons with T2D have an increased risk of micro‐ and macrovascular complications and a 2–4‐fold increased risk of developing cardiovascular disease (CVD). Despite improved treatment opportunities, many persons with T2D do not achieve good metabolic control, increasing their CVD risk (Yang et al. 2021). Within primary healthcare, effective CVD risk communication in T2D is essential. HCPs play a crucial role in promoting healthy lifestyles (ADA 2026). However, research indicates discrepancies between HCPs' and patients' understanding of CVD risk and self‐management, suggesting that risk communication in this context is often complex (Huntink et al. 2015).

## 2 Background

How HCPs communicate risk to persons with T2D has been shown to influence patients' attitudes towards self‐management and medication (Schoulberg et al. 2022). Although many HCPs provide CVD risk information, several studies suggest that opportunities for dialogue and shared interpretation are not always in accordance (Gidlow et al. 2021; Schoulberg et al. 2022).

Risk assessment tools are often used primarily to support medical monitoring rather than to facilitate person‐centred communication (Gidlow et al. 2021). The way CVD risk is communicated also shapes patients' perceptions and their willingness to engage in self‐management behaviours (Yao et al. 2021). A person‐centred, caring and dialogical approach that encourages patients to speak freely and balances negative and positive perspectives appears essential for meaningful risk communication (Britten et al. 2020).

Despite expectations that HCPs provide CVD risk communication and self‐management support (ADA 2026), some persons with T2D report not receiving risk information and not understanding that self‐management activities aim to reduce CVD risk (Jutterström et al. 2024). Among persons with T2D who have experienced a myocardial infarction, some did not perceive themselves as predisposed to CVD prior to the event and did not associate the occurrence of myocardial infarction with their diabetes (Ängerud et al. 2015).

Understanding patients' expectations is important because it shapes the risk‐communication demands placed on HCPs. Many patients wish to participate actively in decision‐making and to discuss individual risk factors rather than receive general advice (McSharry et al. 2020; The Agency for Health and Care Analysis 2021). However, translating population‐based risk estimates into personally meaningful information remains challenging, even when visualisation techniques are used (Huntink et al. 2015; Schoulberg et al. 2022). Visual tools may support understanding, but they can also evoke emotional reactions that influence how risk is perceived (Plant et al. 2022).

Within primary healthcare, person‐centred care (PCC) provides an overarching framework for understanding risk communication, emphasising partnership, dialogue and shared decision‐making (Britten et al. 2020). Strengthening diabetes specialist nurses' capacity to deliver PCC is considered necessary, and effective CVD risk communication is positioned as a core component of PCC (Dautovic et al. 2025; Britten et al. 2020). Although research consistently shows that CVD risk communication is complex and variable in practice, little is known about how HCPs, particularly diabetes specialist nurses, interpret, prioritise, and navigate these conversations within the organisational and relational conditions of everyday work. Existing studies tend to describe communication challenges rather than examine how HCPs themselves make sense of and manage them.

To address this gap, this study explores how cardiovascular risk communication is constructed, negotiated and enacted in practice to persons with type 2 diabetes. Focusing on HCPs' perspectives may contribute new knowledge about the practical, relational and contextual conditions that influence risk communication and offer insights to support more effective, person‐centred strategies in diabetes care.

## 3 The Study

### 3.1 Aim

To explore how cardiovascular risk communication is constructed, negotiated and enacted in practice to persons with type 2 diabetes.

## 4 Methods

### 4.1 Design

The study is empirical, with a qualitative design based on the constructivist approach to the grounded theory method GTM (Charmaz 2014). Empirical data were collected and analysed concurrently using constant comparison and in an iterative process to create an illustrative theory (Charmaz 2014). Reporting of the qualitative study followed the Consolidated Criteria for Reporting Qualitative Research (COREQ) (File S1).

### 4.2 Setting and Recruitment

The project was conducted with diabetes specialist nurses and physicians from nine primary healthcare centres (PHCs) in northern Sweden, covering both urban and sparsely populated areas. These centres differed in size, the number of HCPs, and the patients' access to specialised diabetes follow‐up. Differences in organisational conditions were considered as an important contextual factor influencing both the delivery of diabetes care and participants' experiences of CVD risk communication.

As shown in Table 1, the participants included diabetes specialist nurses and physicians who care for patients with T2D and who had at least 0.5 years of work experience in the field. The executive managers of the PHCs granted permission to conduct the study and identified potential participants. Using convenience sampling, 5 HCPs working with persons with T2D were recruited; thereafter, additional participants were recruited through snowball sampling. A total of 14 individual interviews were conducted, while 7 declined. The sampling was not governed by a strict theory‐driven (theoretical) sampling due to difficulties in recruiting participants. Data collection and analysis proceeded in parallel, with each step informed by previously analysed insights.

**TABLE 1:** Characteristics of the participants in the study.

Characteristics	Participants
Primary healthcare centres	9
Gender	
Male	6
Female	8
Age (years), median (range)	43 (35–56)
Healthcare professionals	14
Diabetes specialist nurses	8
Physicians	6
Professional experience median (years) (range)	7 (0.5–25)

### 4.3 Data Collection

The individual interviews took place during 2022–2023 and were conducted via digital media and audio‐recorded by the first and last authors. The semi‐structured interviews were conducted using an interview guide that gradually evolved as new requirements, ideas, and insights emerged during data collection and analysis (Charmaz 2014). Examples of questions in the interview guide were ‘When do you bring up the risk of developing complications and CVD risk with your patients?’; ‘What complications do you usually talk about?’; ‘Are there complications that you avoid talking about?’; ‘How do you evaluate if a patient has an increased CVD risk?’ What do you think is essential for a patient to change in their lifestyle?’ and ‘What can start such a process?’ The interview guide included follow‐up questions to deepen understanding of concepts that emerged from initial coding (Charmaz 2014). The initial interviews were analysed continuously to identify preliminary categories related to the communication of CVD risk. From previous interviews, we constantly explored variations and more detailed perceptions of the CVD risk, the tools used and perceived barriers to CVD risk communication.

The interviews also included vignettes, which are hypothetical cases used to facilitate discussion and reflection, encouraging participants to reason their way to an optimal and realistic assessment of the cases, as well as how the potential CVD risk could be communicated. Hypothetical cases are used because they are perceived as less threatening than asking HCPs for their opinions or experiences in handling similar situations (Sheringham et al. 2021). The use of vignettes can explain variations in care that are not possible using real‐life data and is an important methodological tool for identifying how unwarranted variations in care can arise (Sheringham et al. 2021). The vignette data, together with interview data, were integrated into the analysis through the transcriptions and line‐by‐line coding (Charmaz 2014). The vignettes provided standardised information, ensuring that all participants assessed the same fundamental information. Questions based on vignettes about CVD risk were also used to clarify HCPs' perceptions and actions regarding CVD risk in relation to patient cases. Examples of vignette questions included ‘How do you consider the patient's risk of CVD, comparing patient 1 and patient 2?’; ‘Explain what you would like to communicate in the meeting with this patient in terms of CVD risk?’; ‘How would you communicate the patient's CVD risk with the patients?’; ‘How do you evaluate the person's willingness and motivation to change to reduce the risk of CVD?’; ‘How do you increase motivation for improved self‐management to reduce the risk of CVD?’; and, ‘How do you cause the patients who are not motivated to change to reduce the risk of CVD?’. The interviews lasted 45–60 min and were transcribed verbatim by the first author.

### 4.4 Data Analysis

The process of interviewing, coding and categorising was conducted using a constant comparative and iterative approach (Charmaz 2014), in which new data were continuously compared with existing codes and categories. Each interview was transcribed verbatim shortly after it took place and MAXQDA software (MAXQDA 2023) was used for the initial coding, which began as soon as rich, detailed data were available. Text that corresponded to the aim was labelled with codes close to the data, using line‐by‐line coding. During the coding process, these codes were continually compared for their similarities and differences. To deepen the analysis during the focused coding, data from MAXQDA were transferred to Post‐it notes, which were then sorted and grouped into subcategories and preliminary categories by identifying patterns and relationships. The theoretical coding involved clarifying the relationships among categories and constructing the core category (Charmaz 2014). During that phase, the analysis was guided by central grounded theory questions like ‘What is happening here?’ ‘What are the basic social processes?’ and ‘How do participants' actions construct them?’. Transparency was ensured by clearly describing how initial codes developed into conceptual categories and how the iterative relationship between data collection and analysis shaped the study's results.

Throughout the analysis, memos were kept with reflections, analytical ideas and questions related to relevant codes and data. The memos contributed to deepening understanding, making the researcher's assumptions visible and strengthening reflexivity.

Theoretical saturation was achieved when no new insights into categories emerged from the data, the categories were conceptually robust and strengthened the theoretical understanding, and when the interviews revealed no additional information (Charmaz 2014). Through theoretical coding, the core category ‘Striving to enhance cardiovascular risk awareness in persons with type 2 diabetes while balancing responsibility and uncertainty’ emerged, summarising the overall model demonstrated by the participants' stories. The theoretical sufficiency was determined through an iterative process of data collection and analysis.

### 4.5 Ethical Considerations

This research was planned, conducted and reported in accordance with the Declaration of Helsinki (WMA). Ethical approval was granted by the Swedish Ethical Review Authority, 2018‐12‐11. The clinical director and the head of the department at the primary health care centres approved the study. Before providing written and verbal consent, participants were informed in writing and verbally and had the opportunity to ask questions. Participation was voluntary.

### 4.6 Rigour

According to Charmaz (2014), trustworthiness can be ensured through credibility, originality, resonance and usefulness. Credibility was strengthened by clearly describing the methodology, analysis procedures and context, including relevant participant characteristics. Memos were generated throughout the analysis to demonstrate the process's dependability. Originality was demonstrated through a novel conceptual interpretation of the data, while resonance was achieved by presenting clear categories supported by illustrative quotes.

A reflexive stance was maintained throughout, with researchers continuously examining how their professional backgrounds and preconceptions might influence their interpretations and reviewing these to reduce bias. The multidisciplinary research team comprising physicians and nurses with expertise in T2D, cardiovascular care, primary healthcare, endocrinological care and intensive care strengthened the analysis through diverse perspectives, critical discussions and regular validation. This created a critical, transparent analytical environment where multiple perspectives were integrated. During interviews, researchers built trust, clarified the study's purpose and encouraged dialogue with participants, enhancing credibility. The reflexive approach increased transparency and supported a robust analysis grounded in the reality of HCPs. The authors present findings, to the best of their knowledge, that have not been previously reported and that are helpful for patients as well as HCPs in primary healthcare and other care contexts. Our study illustrates the importance of grounding the theory in the data and presents participants' real experiences, demonstrating the study's robustness. The authors sorted, reduced and justified their data throughout the process to capture the full scope of the experience under study. To ensure usefulness, the analysis offers interpretations that participants can apply in their everyday work and in other healthcare contexts. The coding and analysis process was regularly discussed in the research team.

## 5 Findings

### 5.1 Participants

Eight diabetes nurses and six physicians working at different PHCs in both rural and urban areas participated. Participants were aged 35–56 years (median 43 years), and their work experiences varied from 0.5 to 25 years.

### 5.2 Core Category, Categories and Subcategories

Through the analysis, four categories and nine subcategories emerged, all connected to the core category ‘Striving to enhance cardiovascular risk awareness in persons with type 2 diabetes while balancing responsibility and uncertainty’ (Table 2). The categories and subcategories are described below and exemplified with original quotes from navigating a complex assessment of cardiovascular risk, struggling to communicate cardiovascular risk in a pedagogical way, trying to build a trusting relationship and simultaneously highlighting disease severity, experiencing loneliness and requesting organisational changes.

**TABLE 2:** Overview of core category, categories and subcategories.

Core category	Category	Subcategory
Striving to enhance cardiovascular risk awareness in persons with type 2 diabetes while balancing responsibility and uncertainty	Navigating a complex assessment of cardiovascular risk	Monitoring HbA1c, lifestyle habits and other risk parameters Using several tools for risk assessment
	Struggling to communicate cardiovascular risk in a pedagogical way	Identifying the patient's understanding Using visual and metaphorical techniques Accepting not reaching out to all patients
	Trying to build a trusting relationship and simultaneously highlighting the disease severity	Creating trust by being personal Balancing between encouraging and demanding
	Experiencing loneliness and requesting organisational changes	Feeling alone and struggling with a lack of shared responsibilities and medical support Striving for a functional team and consensus in diabetes care

#### 5.2.1 Striving to Enhance Cardiovascular Risk Awareness in Persons With Type 2 Diabetes While Balancing Responsibility and Uncertainty

**FIGURE 1:**
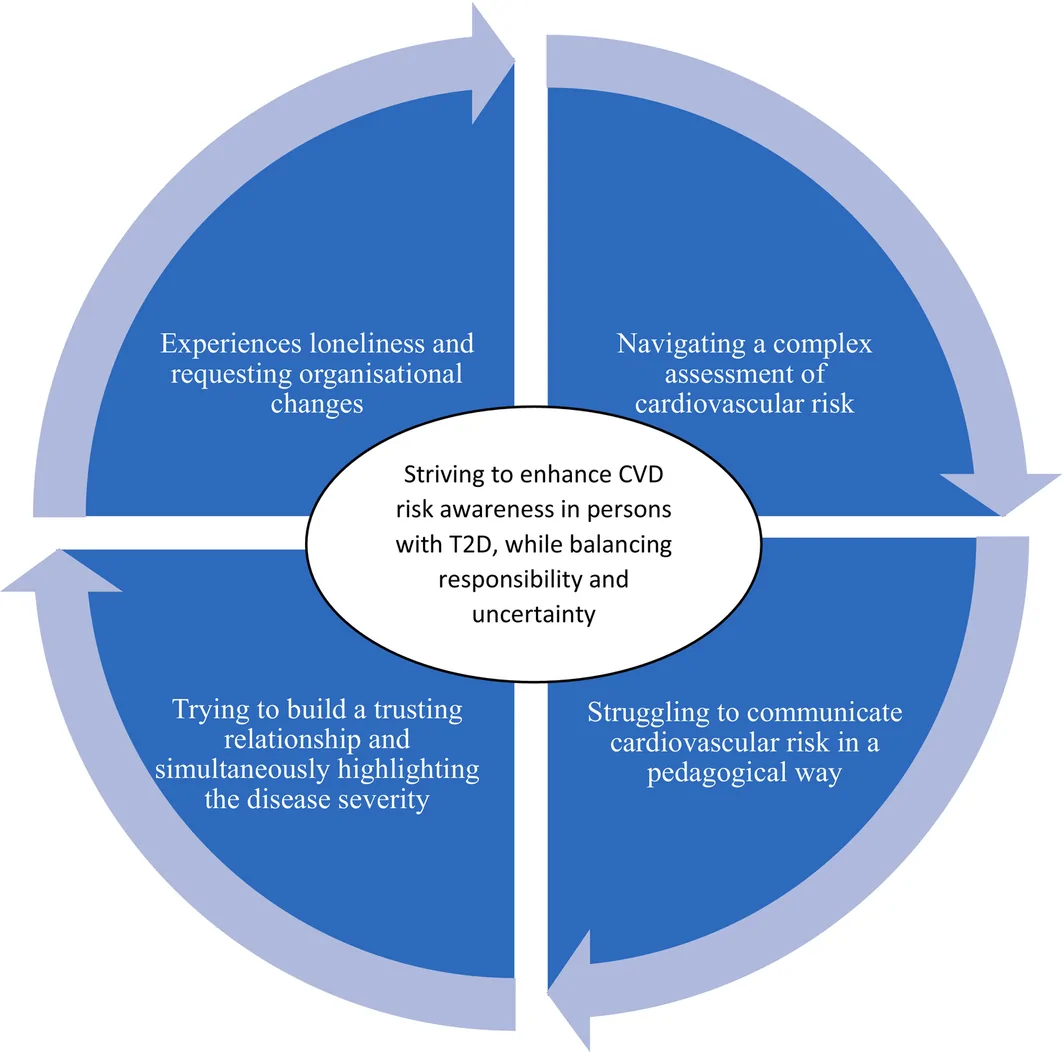
A model describing HCPs' CVD risk communication to persons with type 2 diabetes.

The core category highlighted that the HCPs reported striving to enhance cardiovascular risk awareness in persons with type 2 diabetes while balancing responsibility and uncertainty. CVD risk communication appeared to be relationship‐dependent, characterised by a lack of teamwork, uncertainty and organisational barriers such as limited time and continuity. Furthermore, the core category suggests that Diabetes specialist nurses experienced loneliness, limited support from physicians and the organisation, with insufficient resources, including time constraints, a lack of continuity and medical support, which contributed to uncertainty when communicating CVD risk. A model was developed to describe the relationship between the core category and the categories (Figure 1).

#### 5.2.2 Navigating a Complex Assessment of Cardiovascular Risk

The HCPs described navigating a complex CVD risk assessment. This category emerged from the subcategories described below and highlights how the HCPs identify and evaluate the CVD risk.

##### 5.2.2.1 Monitoring Hba1C, Lifestyle Habits and Other Risk Parameters

The HCPs described different strategies when they identified and assessed the CVD risk through regular follow‐ups, for example, measuring HbA1c and lipid levels as well as blood pressure. They described different focuses on LDL and HbA1c levels. While some focused only on HbA1C when assessing CVD risk, others prioritised lipids initially and then turned their attention to HbA1c. It was difficult to know which best reflected the CVD risk. Lifestyle habits were evaluated, and they strove to detect complications at an early stage. By conducting measurements repeatedly, they aimed to encourage the patients to reflect upon their blood sugar levels. These assessments not only identified risk but also provided a basis for subsequent CVD risk communication; the results were used to explain and contextualise risk for patients.

And it's partly about analysing the samples thoroughly, and then it's about looking at the entire lifestyle and discussing it. We have smoking, we have blood pressure, we have cholesterol levels, we have physical activity, and we have to ensure that blood sugar is at a good level based on the specific patient. (Participant 1, physician)

They also described that they were satisfied with their own professional knowledge but asked for further training and development to stay up to date in the field. They described that considerations regarding lipid‐lowering medications could arise, with questions like, ‘What applies when initiating a medication?’; ‘What can we expect?’; and ‘What side effects are common?’

##### 5.2.2.2 Using Several Tools for Risk Assessment

The HCPs strove for a well‐informed patient and almost all the HCPs described that the risk assessment tool from the national diabetes register (NDR) was used to evaluate and visualise the individual CVD risk when modifying treatments and lifestyles and it enabled the creation of graphs that highlighted adverse trends. The NDR tool helped them to illustrate how lifestyle choices and daily decisions can impact a patient's metrics. The HCPs stated that it is viewed as less sensitive and clearer to present statistics than to communicate other areas of concern. The physicians used risk assessment instruments and standardised tools, that is, Score 2, to identify whether the CVD risk was high or low.

It is possible to follow more with registers, well or NDR, or we have a new tool called Medrave or primary care quality, which we have barely had time to start using but that seems to be a quite good tool, and every now and then find individuals that have the greatest risk and maybe we are then at risk of losing or something like that. (Participant 5, physician)

The HCPs stated that they adhered to guidelines, which included evaluating CVD risk and selecting the appropriate medications to reduce this risk. However, some HCPs refrained from assessing CVD risk because it was perceived as time‐consuming and cumbersome to navigate various systems, resulting in that CVD risk is inadequate communicated.

#### 5.2.3 Struggling to Communicate Cardiovascular Risk in a Pedagogical Way

This category emerged from the subcategories described below and highlights how HCPs attempted to communicate CVD risk using a pedagogical approach, closely linked to the initial assessment of CVD risk, as communication strategies are shaped by how risk is understood.

##### 5.2.3.1 Identifying the Patient's Understanding

The HCPs described that they tried to identify the patient's understanding and knowledge of the disease and their CVD risk by thoroughly asking the individuals to describe why they think they are attending the visit and what the background of the disease is. They further stated that they assessed what information the patient had personally reviewed, including concerns or expectations regarding the visit and the diagnosis, and shaped how HCPs adapted their CVD risk communication strategies.

It was described that the patient's own experiences of having relatives with diabetes who have had heart failure or stroke, or if the patients themselves have suffered complications, could affect insight and the ability to understand and receive information. It was also stated that when they identified a complication in a patient, they explained how diabetes had contributed. However, the HCPs noted that many patients had already drawn this conclusion themselves.

They may have had a relative who has had diabetes who had a heart attack and stroke and visual impairment and all sorts of things, so they have a bit more insight. (Participant 7, diabetes nurse)

##### 5.2.3.2 Using Visual and Metaphorical Techniques

The HCPs highlighted that they create step‐by‐step visual pictures, adapting and customising specific aspects to suit individual patients. The HCPs stated that they illustrate trends through forms, test results and numerical data to show improvements and deteriorations. The HCPs clarified that they utilise explanatory models to illustrate blood sugar levels and high lipid levels. The HCPs described that the communication of CVD risk depended on which patient they met. They asked follow‐up questions, established a dialogue with the patients, and made inquiries about what the patient comprehended from the information to ensure their understanding of its significance. Some HCPs described that they requested the patient to recount what had been said during the appointment and what changes needed to be accomplished. The HCPs stated that they collaboratively summarised with the patient what they had assembled and what agreements were reached. The HCPs also said that some patients became very motivated to reduce CVD risk, but they also dealt with fear and unwillingness from patients to take medication and perform self‐management activities to reduce CVD risk, even though the same information was given. The HCPs also strove to adapt the medical language and make the language more accessible to the patients.

… to adapt information so that the patients can take it in, because if I give completely wrong adapted information, it's quite a waste, I'm saying that you have an increased risk of vision changes, stroke, heart attack, kidney failure. I'm trying to be quite clear, and you see that some are like that, but at the same time, I think it's very important that you don't wrap it up too much and are too vague and kind (Participant 9, diabetes nurse)

Some HCPs described that they provide a comprehensive explanation from ‘head to toe’ when communicating CVD risk. They find the appropriate approach individually, considering the absence of a structured follow‐up and a template for what should be included in a visit.

Additionally, some of the HCPs said that they communicate microvascular complications and not always macrovascular complications or that they communicate them at different times during the visit. HCPs also described a sense of satisfaction and professional pride when they succeeded in reaching patients, observed that patients understood and absorbed the information, and saw them make lifestyle changes that contributed to improved health and reduced CVD risk.

##### 5.2.3.3 Accepting Not Reaching Out to All Patients

The HCPs described that feelings of frustration could arise during the communication process. They tried to support their patients, but they also expressed feelings of failure when confronted with the reality of not being able to reach certain individuals. In certain occasions, the HCPs felt obliged to acknowledge the necessity of ‘having to give up’ on certain patients who do not follow lifestyle advice and medical treatment. This includes patients who lack motivation for self‐management, those with mental health issues, and those with diminished intellectual capacity. The HCPs explained that they must prioritise patients who are willing to endure some form of change, but they emphasised that this decision is a challenging one.

In that case, I have to focus my efforts on those who are willing to make some sort of change, and I find that decision very difficult to make. (Participant 12, diabetes nurse)

The HCPs described that some patients conveyed feelings of shame and a sense of failure when they did not achieve CVD risk targets and optimal self‐management activities. The HCPs attempted to clarify to the patients that these conditions are influenced by hereditary predispositions and lifestyle‐related factors. However, the HCPs stated that it is difficult to meet the patient's concerns and expectations.

They described that lifestyle change and the establishment of patient compliance must be given time, and if patients do not make progress the first time, they must bring the individual back. However, they underscored that ultimately, it is the patient's life and their choice to make lifestyle changes.

The difficult part is reaching these individuals; perhaps they don't want to acknowledge that they've received a diagnosis or maybe, well, don't see it as a problem and so on. So, what should I say? It becomes a mix of compassionate firmness, scare tactics, and enticing… (Participant 13, diabetes nurse)

HCPs described risk communication related to self‐management as complex and challenging. They expressed feelings of doubt when they felt they were not reaching the patient. They viewed it as a significant responsibility to identify an appropriate way of providing information. They also sometimes questioned their own competence in communicating CVD risk. In addition, HCPs described occasionally avoiding providing information when patients were perceived as fearful, experiencing information overload or dealing with mental health issues.

#### 5.2.4 Trying to Build a Trusting Relationship and Simultaneously Highlighting the Disease Severity

This category emerged from the subcategories described below and highlights the HCP's experiences of building a trusting relationship of care and being supportive. Building trust is closely linked to both CVD risk assessment and risk communication and emphasises the severity of diabetes and the importance of self‐management to reduce CVD risk.

##### 5.2.4.1 Creating Trust and Being Personal

The HCPs described that they strive to build a professional but personal relationship with the patients. They stated that they used their own experiences as a driving force in the meeting and thus became more personal. They described the need to build trust and credibility and an alliance with the patients to create participation that would motivate self‐responsibility, patient activity and empowerment. The HCPs said that they strove to get patients to trust the competence and knowledge they have in their professional role. To achieve this, they described that they needed to get to know the patients, providing advice near the patient's life, demonstrating their competence, and supporting and encouraging them by instilling hope and reinforcing progress.

But this trust again, if you want to accomplish change in people, they must start by trusting you. Um, so building trust over time with the help of continuity is crucial. (Participant 4, physician)

##### 5.2.4.2 Balancing Between Being Encouraging and Demanding

The HCPs reported that several patients did not understand the seriousness of T2D and its associated increased risk of CVD. Therefore, repeated information was considered essential and beneficial in helping patients understand their situation. The HCPs described a need to balance encouragement with demands, emphasising the importance of lifestyle changes and medical treatment to reduce CVD risk. They gave positive feedback to strengthen the patients' understanding, but also clarified the consequences of poor self‐management. They did not want to scare or blame patients but instead aimed to mobilise patients' own resources, although they also acknowledged that a more direct approach was sometimes necessary to motivate change. HCPs described seeking to convey the presence of risks while emphasising that patients could influence their own risk. They stated that they identified the patient's motivation, whether it was related to health concerns, fear or stress, and highlighted the importance of allowing time for reflection. They explained that they linked the currently deteriorating values to diabetes to emphasise the seriousness of CVD risk. Furthermore, HCPs described using patients' own experiences to discern motivational factors and to support individuals' capacity to implement lifestyle changes.

However, they acknowledged that motivating lifestyle changes could be challenging when patients perceive themselves as healthy and therefore sometimes avoided discussing risk.

I like to talk about fears and expectations, to try to find what is a good motivator for the patient as well. So that you kind of don't scare them into lifestyle changes, that's not the goal in any way. I really don't think the patient has any benefit from that, but rather just explain the situation and what we can do to make you feel as well as possible. (Participant 2, physician)

The HCPs emphasised the significance of refraining from blaming patients and highlighted the potential for improvement in all aspects. Some patients, despite being diligent, maintaining a healthy weight, and making lifestyle changes, still encountered significant complications.

#### 5.2.5 Experiencing Loneliness and Requesting Organisational Changes

This category emerged from the subcategories described below and highlights the diabetes specialist nurses' experiences of loneliness and requests medical support and a functional team. HCPs emphasised shared responsibility, continuity and sufficient time to achieve CVD risk communication.

##### 5.2.5.1 Feeling Alone and Struggling With a Lack of Shared Responsibilities and Medical Support

Diabetic specialist nurses described that patients rarely encounter physicians. They described that they felt alone in the overall responsibility for T2D patients and that they often had to took a lot of responsibility for the physician's obligations in the diabetes team due to a physician shortage. They also needed to take a lot of responsibility in the CVD risk communication, information about medical treatment, and other diabetes complications, instead of supporting self‐management activities.

The diabetes specialist nurses described that they take samples, perform a comprehensive assessment, and then escalate it to the physicians when they determine it is necessary. However, HCPs described varying levels of ambition in reducing CVD risk. Physicians and diabetes specialist nurses often apply different strategies to the same patient, particularly regarding how treatment should be delivered, which may result in uncertainty in managing CVD risk.

Some diabetes specialist nurses stated that they trusted the physicians they worked with, but others said they sometimes did not trust certain physicians or the decisions they made.

So it has happened that I have gone to the doctor, not been satisfied when I left, and I have thought that I will wait until next week, because then another doctor (haha) will come, and I will get a better answer (haha), so… (Participant 5, diabetes nurse)

However, some of the physicians saw it as their responsibility to inform the patients about CVD risk, especially in situations of understaffing and lack of knowledge among colleagues. They described that risk was communicated in different ways and they asked for shared responsibility, a common model to work from, a structured way of working and an increased consensus in the diabetes team that communication and finding risk factors should take place. However, they also pronounced an individual responsibility for the risk information.

The HCPs advocated for shared responsibility among physicians, diabetes specialist nurses and other diabetes team professionals. They described a desire for teamwork that did not always exist and for a cohesive working model. One of the HCPs articulated the notion that the team's nonexistence:

When it comes to diabetes patients, they rarely meet with physicians in a planned manner. Instead, almost everything is managed through nurse‐based appointments. In this way, diabetes nurses have increased responsibility for identifying and communicating complications (Participant 3, physician)

The HCPs advocated that there must be an interaction between the diabetes specialist nurse and the physicians, and more diabetic‐focused meetings instead of the regular rounds with the physicians. However, the HCPs also explained that they sometimes felt collegial support and learned from other professionals in the team, through interdisciplinary group discussions, the sharing of pedagogical materials or clinical judgements.

While some HCPs expressed scepticism regarding the team's capacity to sustain continuity in primary care, they characterised a team as a concept requiring integration and regular meetings. Involvement in multiple teams at a healthcare centre was depicted as demanding in terms of time and would detract from patient interactions.

The HCPs also advocated for the development of materials aimed at ensuring consistency in their approach, emphasising that everyone has the right to receive consistent diabetes care regardless of their geographic location. They suggested formulating working strategies for diabetes patients, presenting CVD risk information and establishing effective means of ensuring that the entire team adheres to standardised procedures.

##### 5.2.5.2 Striving for a Functional Diabetes Team and Consensus in Diabetes Care

The HCPs described having few meetings with patients, despite guidelines recommending at least two visits per year. Many patients received only one visit, either from a diabetes nurse or a physician. HCPs reported a lack of continuity among physicians, making it difficult to achieve effective team collaboration. For example, they felt it was difficult to answer follow‐up questions, detect complications and perform a foot examination in a single visit. The HCPs described a lack of coordination that existed, and that they had individually developed their ways of working. They called for better conditions, more time and improved staffing in diabetes care.

The HCPs reported that the diabetes team lacked a structured way of working and a shared understanding of diabetes care, and that no time was allocated to sit together and discuss care. They expressed a desire for a better consensus in diabetes care.

So I think it's very important that we are a team and that we use the same approach, so that I don't talk about blood fats and blood fats and the patient refuses, and then the doctor thinks, yes, that looks good, and just says, no, they don't reach any target values at all, but at a quick glance, they might think it looks good. So it's about everyone being informed and knowing what applies, but I think that yes, they should hear the same information from both sides (Participant 1, diabetes nurse)

HCPs reported that patient accessibility was limited, and, as a result, patients also had limited access to HCPs. Visits became checklist‐oriented and followed a conveyor belt‐like approach. The HCPs' times, they needed to extend the duration of appointments to accommodate this. The opportunity to detect complications early was limited by infrequent patient encounters. They also stated that providing information in an intensive and focused manner could increase patients' understanding of the seriousness of their condition. Continuity fostered greater trust, whereas limited time for follow‐up questions hindered effective risk communication. The HCPs mentioned that they addressed this dilemma by encouraging patients to write down questions for their next visit.

I think that one must, after all, acknowledge that it is a fact that we have limited resources, we have limited time with the patients, and I suppose that many can relate to this: feeling a sense of inadequacy. Um, one would like to do more, have more time to explain or follow up (Participant 5, physician)

## 6 Discussion

This study explored how CVD risk communication is constructed, negotiated and enacted in practice to persons with type 2 diabetes. The core category striving to enhance cardiovascular risk awareness in persons with type 2 diabetes while balancing responsibility and uncertainty illustrates how risk communication can be conceptualised in a model, showing that it is not a simple transfer of information but an ongoing dialogue. Our study also shows that the HCPs feel alone and struggle with a lack of shared responsibilities and medical support, and strive for a functional team and consensus in diabetes care.

Our findings highlight that although the HCPs repeatedly measured parameters and used CVD risk‐assessment tools, HCPs reported that these strategies did not always increase risk awareness or self‐management behaviours. While previous studies emphasise the value of visual and digital risk assessment tools (Goh et al. 2021), our analysis suggests that such tools alone may be insufficient. This helps explain why risk communication is often perceived as inadequate by patients (Jutterström et al. 2024).

HCPs described efforts to engage well‐informed patients, using patients' experiences to enhance CVD risk awareness. This aligns with Ben Chmo et al. (2023), that states that patients' day‐to‐day life responsibilities and interactions with HCPs shape how risk is perceived and managed. HCPs described variation in patients' perceptions of risk, which is consistent with other studies (Saeedi et al. 2020; Ben Chmo et al. 2023). Our study points out how HCPs navigate these variations in real time and experience the tension between objective CVD risk communication and patients' subjective interpretations. This real‐time navigation is a central part of our model, in which HCPs oscillate between navigating CVD risk communication and communicating CVD risk pedagogically (Figure 1). This demonstrates that risk communication is a dynamic rather than a linear transfer of information. Although patients' risk perceptions vary, the HCPs tend to inform rather than communicate CVD risk in a person‐centred manner.

Chu et al. (2023) emphasise the importance of HCPs in raising awareness of CVD risk among patients with T2D. However, HCPs often rely on objective, fact‐based communication, which may overlook patients' emotional responses. Our findings suggest that HCPs need to strengthen skills in addressing emotional and existential concerns. HCPs reported that they tried to use persons with T2D experiences to increase CVD risk awareness, but variations in how patients perceive CVD risk require continuous adaptation. This real‐time balance between objective CVD risk information and subjective interpretations is an important part of the CVD risk process. Our results indicate that the HCPs strove to understand patients' knowledge and to provide person‐centred information, in line with Ekman et al. (2021), who emphasise the importance of informing patients of their personal resources. Although person‐centred communication is often presented as an ideal, many patients with T2D struggle to understand how to manage their diet, medications and lifestyle choices (Chepulis et al. 2023). Our results indicate that HCPs must undertake considerable interpretative work to adapt patients' everyday lives and motivation for self‐management. Person‐centeredness, therefore, involves more than adapting information; it requires creating a shared understanding of what risk means for the individual and actively creating meaning together with the patient.

The results point to the challenge of translating medical content, such as the link between diabetes and CVD risk, into clear, relevant information. Although HCPs feel responsible for CVD risk communication, they are frustrated when they cannot reach all patients. Managing diabetes and heart disease simultaneously requires interdisciplinary collaboration (Suman et al. 2023). Our findings highlight the need for ongoing discussions among HCPs about appropriate strategies, as different approaches may affect patients' understanding of CVD risk in different ways.

HCPs also expressed a desire to convey the seriousness of CVD risk without eliciting feelings of guilt or shame. Studies illustrate that a friendly and reassuring approach can reduce stigma (Rai et al. 2020). Additionally, our findings reveal that HCPs want to avoid frightening patients, yet some individuals may not grasp the severity of the risk until an event occurs. This reveals an ethical tension between professional responsibility and the desire to protect patients from fear. Previous research shows that when a patient expresses shame, HCPs need to address these feelings before moving on (Solomon et al. 2022). Helping patients to articulate their emotions can strengthen the relationship and improve communication. Our results indicate that HCPs strive for a professional yet personal approach. Trust and support are central to treatment adherence (Ben Chmo et al. 2023), and poor communication increases the risk of low compliance. The ability to provide support without judgement is therefore crucial for building trust and facilitating lifestyle changes (Lönnberg et al. 2020). It is advantageous for CVD risk communication to be based on a stable relationship between HCPs and patients. Professional knowledge, empathy and relationship‐building skills facilitate CVD risk communication. HCPs described that professionalism was important; however, the literature states that it is often necessary to consider the patient's individual views and emotional aspects to connect with persons with type 2 diabetes (Britten et al. 2020).

HCPs emphasised the importance of adapting to patients' varying abilities to understand risk information, which can be affected by factors such as lack of motivation, cognitive impairment, mental health conditions and language barriers. In line with Chepulis et al. (2023), our results show that education and language shape CVD risk communication. One conclusion is that a patient's lack of interest or unwillingness may be attributable to other factors, such as shortcomings in the approach, time constraints or HCPs' attitudes.

Under constant pressure to meet healthcare expectations and provide person‐centred care, HCPs describe limited time, limited continuity and a lack of organisational support as barriers to effective CVD risk communication. While resource constraints affect diabetes care (Tan et al. 2020), our study illustrates how these structural factors shape the communication process itself. When HCPs lack time for follow‐up or when team structures are unclear, the ability to build the trust required for effective risk communication is compromised. This demonstrates that risk communication challenges are not only individual but also organisational and systemic.

Collaboration among HCPs regarding persons with T2D was described as insufficient. Some HCPs sought shared responsibility and multidisciplinary teamwork, but collaboration was constrained by HCP shortages and inadequate routines. Multidisciplinary teams improve diabetes care and prevent complications (Desse et al. 2023; Tan et al. 2020), yet inconsistent information‐sharing limits patients' access to available expertise. The shortage of HCPs in primary healthcare leads to loneliness, uncertainty, frustration and limits the ability to provide comprehensive care. Limited access to healthcare increases the risk of complications (Ben Chmo et al. 2023). Our model emphasises the importance of assessing and communicating CVD risk, highlighting the seriousness without frightening the patients, and managing uncertainty in the face of limited support during time constraints, lack of continuity and an inadequately functioning diabetes team.

This study shows that HCPs aspire to communicate CVD risk through close, intensive engagement but provide insufficient follow‐up and continuity, undermining effective risk communication.

The theoretical contribution lies in illustrating how HCPs navigate between medical knowledge, patients' subjective interpretations and structural limitations. In practice, the findings highlight the need to support HCPs' ability to listen to patients' narratives and ask in‐depth questions about how they perceive living with T2D and their personal CVD risk. Ekman et al. (2021) report that storytelling places experience and understanding in a context and leads to an increased understanding of the illness and helps to connect the parts into a whole, for example, that one's actions affect the risk, which can enhance the diabetes specialist nurse's understanding of how they can better support patients individually, which is in line with person‐centred care (Britten et al. 2020).

Our results highlight a nuanced understanding of why CVD risk communication to enhance risk awareness is complex and sometimes difficult to establish.

### 6.1 Strengths and Limitations of the Work

The empirical data were collected and analysed concurrently through constant comparison, in an iterative process in which new data were compared with previously collected material. This approach strengthened analytical depth and supported the development of grounded interpretations.

A total of 14 HCPs, comprising six physicians and eight diabetes nurses, participated in the study. Recruiting participants for this study was challenging. Some HCPs stated that they were not permitted to participate during working hours and did not wish to do so outside working hours. This may have contributed to selection bias, as those who agreed to participate might have been more comfortable with or interested in the topic. Consequently, the sample may not fully reflect the range of perspectives among HCPs. However, the participants represented nine healthcare centres across both rural and urban areas and varied in age and professional experience, contributing to heterogeneity in the dataset.

Due to recruitment challenges, snowball sampling was used. While this method facilitated access to participants, it also carries well‐known limitations, including potential bias and limited representativeness, as it relies on participants' social networks. However, through snowball sampling, we recruited participants so that theoretical sufficiency was achieved.

The researchers' professional experiences and interpretations shaped the study in several ways. Their familiarity with clinical roles and contexts informed nuanced interpretations of the narratives. However, their professional experiences may also have introduced preconceptions that could influence data interpretation. Reflexivity was therefore essential throughout the research process.

Rich data were collected through digital, in‐depth interviews. This mode of data collection may have limited rapport and non‐verbal communication. At the same time, the digital format reduced contextual biases and enabled participants to engage from familiar environments, which may have supported openness.

The interview guide included vignettes (hypothetical cases). These vignettes were used to understand HCPs' perceptions, judgements and intentions regarding T2D and CVD risk. For instance, they were used to explore how HCPs would communicate CVD risk and how they thought patients would respond to certain communication. These vignettes allowed the researchers to explore ethically sensitive or complex situations in a controlled manner.

Most participants were female, which may have influenced the findings; a more balanced gender distribution could have yielded additional perspectives. Despite variations in age, sex and experience, similar topics emerged across interviews, suggesting a degree of consistency in the narratives.

Several authors contributed to the analysis, bringing multiple interpretive perspectives and enhancing the credibility of the findings. The use of memos supported the analytic process by helping the researchers articulate emerging ideas and reflect on how interpretations evolved over time.

### 6.2 Recommendations for Further Research

More research is needed in which persons with T2D and HCPs explore collaboratively, on equal terms, based on their diverse knowledge and experiences, how the CVD risk should be communicated. Intervention studies aimed at supporting and assessing risk awareness, improving communication regarding CVD risk, and effectively motivating and supporting these individuals in enhanced self‐care activities are also necessary.

### 6.3 Implications for Policy and Practice

HCPs often struggle to communicate effectively with patients about how their actions can increase their CVD risk. It is crucial to recognise the pivotal role of HCPs in this communication process and to ensure that they possess the necessary resources to deliver high‐quality care. However, there remains a knowledge gap regarding how to effectively communicate CVD risk. It is unclear whether the solution lies in new educational approaches, the interaction process or other factors such as person‐centred care. Our study found that HCPs find it difficult to communicate CVD risk information to patients with T2D in a way that enables patients to articulate their understanding of and views on their risk. Therefore, our study plays a central role in highlighting the challenges of communicating CVD risk in primary healthcare settings.

## 7 Conclusion

HCPs feel a responsibility to communicate CVD risk but simultaneously experience uncertainty about how to assess patients' individual CVD risk. They also describe difficulties in discussing the CVD risk associated with the seriousness of T2D without making the message either too vague or overly demanding. At the same time, they find it difficult to determine whether patients have actually understood the risk information. Diabetes specialist nurses further describe feelings of professional isolation, limited medical support from physicians, and a lack of shared responsibility within the diabetes care team. The HCPs requested organisational resources such as time and continuity. Taken together, these challenges require HCPs to balance their responsibility to communicate CVD risk with the uncertainty they experience regarding both risk assessment and whether the information is received and understood by patients. Future research should explore how CVD risk communication can be enacted in ways that avoid vagueness or threat, while still supporting meaningful patient engagement and self‐management.

## Author Contributions

All authors conceptualised the study, interpreted the results and revised the draft. The study was designed by A.‐L.S., L.J. and K.H.Ä., A.‐L.S., L.J. and J.O. contributed to participant recruitment. A.‐L.S., L.J. conducted the interviews and transcriptions. A.‐L.S., L.J. and K.H.Ä. performed the coding and analysis. A.‐L.S. drafted the first version of the manuscript, and all authors provided critical revisions and read and approved the final version.

## Funding

The study is funded by a regional agreement between Umeå University and Region Västerbotten (ALF; RV‐981327), the Healthcare Region of Northern Sweden (VISARE NORR), the Swedish Diabetes Foundation (DIA2022‐748), the Rönnbäret Scholarship and the Medical Foundation in Skellefteå.

## Ethics Statement

This research was planned, conducted and reported in accordance with the Declaration of Helsinki (WMA). The clinical director and the head of the department at the primary health care centres provided approval that the study could be conducted. Before the interview, the participants were informed about the study both verbally and in writing and had the opportunity to ask questions. The participation was voluntary. The information included the study's aim, participation, the right to withdraw and participant confidentiality. Ethical approval was granted by the regional review board (No. 2018/445‐31).

## Conflicts of Interest

The authors declare no conflicts of interest.

## Supporting information


**File S1:** COREQ (COnsolidated criteria for REporting Qualitative research) Checklist.

## Data Availability

The data that support the findings of this study are available on request from the corresponding author. The data are not publicly available due to privacy or ethical restrictions.
